# The Best of Both Worlds: Eliminating Creatine Kinase-Muscle/Brain (CK-MB) Testing in the Emergency Department Leads to Lower Costs Without Missed Clinical Diagnoses

**DOI:** 10.7759/cureus.15150

**Published:** 2021-05-21

**Authors:** Prajna A Sahadeo, Akiva A Dym, Luke B Berry, Pegah Bahar, Arnav Singla, Melissa Cheta, Rohan Bhansali, Sean LaVine, Jordan Laser, Mark Richman

**Affiliations:** 1 Emergency Medicine, Northwell Health Long Island Jewish Medical Center, Long Island, USA; 2 Emergency Medicine, Rutgers New Jersey Medical School, Newark, USA; 3 Emergency Medicine, University of Incarnate Word School of Osteopathic Medicine, San Antonio, USA; 4 Cardiology, Northwell Health Long Island Jewish Medical Center, Long Island, USA; 5 Internal Medicine, Northwell Health Long Island Jewish Medical Center, Long Island, USA; 6 Pathology and Laboratory Medicine, Donald and Barbara Zucker School of Medicine at Hofstra/Northwell, Hempstead, USA

**Keywords:** ck-mb, cardiac troponin, creatine kinase, acute coronary syndrome, myocardial infarction, unstable angina, troponin

## Abstract

The 2014 American Heart Association/American College of Cardiology (AHA/ACC) clinical guidelines recommend cardiac troponin as a superior biomarker to creatine kinase (CK) and creatine kinase-muscle/brain (CK-MB) for the detection of acute coronary syndrome (ACS), namely myocardial infarction and unstable angina. In April 2018, our Emergency Department (ED) transitioned from using standard troponin to using high-sensitivity troponin T, and adopted a clinical guideline consistent with the AHA/ACC. The guideline recommended high-sensitivity troponin T without CK/CK-MB testing in the majority of clinical situations, limiting CK/CK-MB testing to two specific clinical cases: 1) estimated glomerular filtration rate (eGFR) value <15 mL/min, or 2) recent acute coronary syndrome (ACS) event. Per our ED’s policy, a “negative” troponin T was defined as being below the limit of detection (LOD) (i.e., <6 ng/L); such a value obtained at least 3 hours after symptom onset “ruled out” an ACS event and did not require a repeat troponin.

The goal of this retrospective study was to determine whether the guideline limiting CK-MB testing missed clinically-significant cardiac outcomes (ACS or new diagnosis of coronary artery disease [CAD]) or was associated with mortality. Pre-implementation data (July 1, 2017 - December 31, 2017) was compared with post-implementation data (July 1, 2018 - December 31, 2018). After guideline introduction, CK/CK-MB ordering decreased by nearly 90%, while troponin ordering increased by nearly 20%, likely due to the introduction in June 2018 of high-sensitivity troponin T, which yielded numerous intermediate/indeterminate-range results that prompted repeat testing. Fewer than 1.5% of patients with a “negative” troponin (below the LOD) and a “positive” CK-MB (above the upper limit of normal [ULN]) had ACS or new-diagnosis CAD; patients with either diagnosis did not expire during their hospital stay or within 30 days of their index visit. CK-MB Index, which has a higher specificity than CK, only found ACS or new CAD among 0.8% of positive results. Considering both decreased CK/CK-MB and increased troponin ordering, the net annual direct cost savings in cardiac biomarker testing was extrapolated to $12,700. Had our institution not transitioned to higher cost high-sensitivity troponin ($2.054/unit) from standard troponin ($1.65/unit), and had the rate of troponin ordering increased solely proportionate to the rate of ED visit increase (2% year-over-year) rather than increase nearly 20% (likely due to the transition to high-sensitivity troponin), then the total six-month direct costs on troponin testing would have been $14,632 instead of $21,267.12, and annual direct cost savings would have been $18,945.80 instead of $12,700. The new ED clinical guideline did not result in a significant number of missed ACS or new-diagnosis CAD, and was associated with direct cost savings. These savings probably underestimate total savings, as the reduced number of “false-positive” CK-MB results likely prevented additional costs, such as hospitalization, specialty consultation, coronary calcium CT, echocardiogram, cardiac stress test, and coronary artery catheterization.

## Introduction

The diagnosis of acute coronary syndrome (ACS), including acute myocardial infarction (AMI) and unstable angina, can be suggested through serologic testing of myoglobin, creatine kinase (CK), and creatine kinase-muscle/brain (CK-MB), CK-MB Index (CK-MB divided by total CK), and, more recently, troponin C, I, or T. Compared with CK/CK-MB and CK-MB Index, troponin levels more reliably rise in ACS. Troponin has several advantages over myoglobin, CK/CK-MB, and CK-MB Index. In AMI, myoglobin levels rise rapidly (within two hours), peak in 10 hours, and fall quickly (1-2 days). CK-MB levels rise more slowly (3-12 hours), peaking in 24 hours, and remaining elevated two-three days. Troponin levels rise within 3-12 hours, peak in 24-48 hours, and decrease over 5-14 days, providing a longer period of time in detecting AMI [1]. High-sensitivity troponin T is more sensitive than CK-MB (98.7% [2] vs. 90% [1]). CK-MB Index has a sensitivity of 46.9% and specificity of 96.1% for AMI and sensitivity of 23.5% and specificity of 96.1% for other ACS [1]. 

A study by Volz et al. analyzed over 11,000 patient encounters where cardiac biomarkers were ordered and found zero cases in which a patient was found to have had AMI when the CK-MB was positive and troponin negative [3]. In 2014, the American Heart Association/American College of Cardiology (AHA/ACC) recommended cardiac troponin as a superior biomarker to myoglobin and CK/CK-MB [1].

Troponin levels exist as a spectrum, with likelihood and severity of myocardial damage proportionate to troponin level. However, for operational purposes, organizations have adopted models that are either binary (“negative” or "positive/elevated") or with three categories: "negative," "intermediate/indeterminate," and "positive/elevated [4]." At our institution, a troponin T level that was undetectable/below the limit of detection (<6 ng/L) was considered "negative;" values between the limit of detection (LOD) (6 ng/L) and 51 ng/L were considered "intermediate/indeterminate;" and values >51 ng/L were considered "positive/elevated," consistent with the European Society of Cardiology’s definition of what constitutes a “high” value for our institution's high-sensitivity troponin T assay (Elecsys Troponin T, electrochemiluminescence immunoassay by Roche Diagnostics, Rotkreuz, Switzerland) [5]. 

In April 2018, our Emergency Department (ED) created and adopted a clinical guideline consistent with the AHA/ACC guidelines, switching from standard troponin (Elecsys Troponin T, 4th generation) to troponin T (Elecsys Troponin T, electrochemiluminescence immunoassay by Roche) without CK/CK-MB (Roche CK-MB by electrochemiluminescence immunoassay) testing in the majority of clinical situations. Per our ED’s policy, a “negative” troponin T was defined as being below LOD (<6 ng/L); such a value obtained at least three hours after symptom onset “ruled out” ACS and did not require a repeat troponin. This policy was based on ED literature [2,6,7] and was more conservative than that recommended by the Agency for Healthcare Research and Quality’s Effective Health Care Program and the National Academy of Clinical Biochemistry Laboratory Medicine, which suggest that values below the 99th percentile (not below the LOD) represent a “negative” troponin, and therefore, unlikely ACS [8]. The policy further stated that results between the LOD and the 99th percentile (i.e., between 6-14 ng/L) were within the intermediate/indeterminate range (6-51 ng/L). Such results could not be considered “negative” and mandated a repeat troponin at least 1 hour after the initial troponin was drawn. Consistent with the AHA/ACC, a change (“delta”) of ≥20% suggested ACS, whereas stable troponin values suggested a chronic cardiac (e.g., congestive heart failure) or non-ischemic (e.g., renal failure) [1]. As per the European Society of Cardiology, values >51 ng/L were considered “elevated.” [5]

In the new guideline, CK/CK-MB testing was limited to two specific clinical cases: 1) renal failure with an estimated glomerular filtration rate (eGFR) value <15 mL/min, and 2) if the patient had a known or suspected ACS within the previous two weeks. The exception for renal failure was because troponin is elevated in 100% of dialysis patients without active myocardial injury, whereas CK-MB is elevated in only 30-50% of such patients. An elevated CK-MB in a dialysis patient, therefore, more likely indicates an acute event [9]. The exception for recent ACS was since troponin remains elevated 5-14 days, an elevated CK/CK-MB (with its shorter serum lifespan) can help distinguish whether an elevated troponin level represents a new ACS event (in which case, CK-MB would likely also be elevated) versus a resolving recent ACS event (in which case, CK-MB would be within normal limits).

The aims of this study were: 1) determine the percentage of patients in a large academic hospital with a largely under-served and non-English speaking population in whom the elimination of CK-MB testing would potentially have missed ACS events; and 2) determine whether implementation of the guideline to reduce CK-MB testing led to direct cost-savings.

## Materials and methods

We conducted a retrospective chart review of patients with a visit between July 1, 2017 to December 31, 2017 and July 1, 2018 to December 31, 2018 to an academic, urban, tertiary-care ED with an approximate annual volume of 100,000 adult visits. In April 2018, our ED adopted the AHA/ACC guidelines for use of troponin instead of CK-MB, and in June 2018, we implemented high-sensitivity troponin T. All adult patients with joint troponin and CK-MB orders placed in the ED in these time periods were included in the sample. The final sample for retrospective chart review included patients with a “negative” troponin T (below the LOD [<6 ng/L], as per our ED’s protocol) and “positive” CK-MB (above the upper limit of normal [ULN] [> 6.6 ng/mL in males, >4.7 ng/mL in females at our institution]). Demographic information; disposition; chief complaint; past medical history; troponin and CK-MB results; whether a stress test or cardiac catheterization was conducted; and whether there were any clinically significant cardiac outcomes (diagnosis of ACS, AMI, or unstable angina) were recorded during chart review. The main outcome was pre-defined as a new diagnosis of acute coronary syndrome (ACS) or coronary artery disease (CAD) during a patient’s ED/hospital visit, as determined by chart review. Patients without a history of ACS or CAD prior to the index ED/hospital visit, but whose index visit discharge summary indicated ACS or CAD, were considered to have new-onset ACS or CAD. Patients discharged from the ED were followed for 30 days post-ED discharge. Charts were reviewed for up to 30 days post-discharge from index visit to determine whether an ACS event had occurred during this timeframe; authors looked at ED and hospital discharge diagnoses to assess whether an ACS event had occurred. We excluded from analysis patients with clinically obvious ACS (e.g., ST-elevation myocardial infarction or cardiac arrest), as cardiac biomarker testing would not affect diagnosis or disposition. CK-MB Index was also evaluated for all patients, with a positive value being >2.5. Finally, we analyzed the absolute change in the number of troponin and CK/CK-MB lab tests ordered before and after implementation of the new ED guideline regarding CK/CK-MB testing and calculated changes in direct lab test costs to the institution.

The pre-implementation period was the six-month period from July 1, 2017 through December 31, 2017, with the post-implementation period the six-month period from July 1, 2018 through December 31, 2018; troponin T was introduced in June 2018, shortly before the post-implementation period. These periods of time were coordinated to minimize any seasonal impact on testing patterns, patient volume, and house staff progression through their training. In acknowledgement that it often takes time to acclimate to new guidelines, we set the start date for post-implementation analysis three months after guideline introduction (i.e., July 1, 2018, rather than April 2018 [when the guideline was released]). Excluded from analysis were patients with eGFR value <15 mL/min (per the ED guideline, CK/CK-MB could still be ordered on this subset of patients). Per-unit cost of high-sensitivity troponin T ($2.054) and CK-MB orders ($1.054) were compared from 2017 pre-implementation period to the 2018 post-implementation period to assess for the potential direct financial savings associated with guideline implementation. As the transition from standard troponin to high-sensitivity troponin in June 2018 was planned well in advance, and independent of the CK-MB testing guidelines, we did not include in the financial estimates the costs of staffing and equipment for the new assay. A Chi-square analysis was used to determine significance for guideline adherence, as indicated by reduction in CK/CK- MB ordering.

This study was deemed exempt by the Institutional Review Board as a quality improvement project to gauge compliance with and unintended consequences of transitioning to meet the standard of care for laboratory testing for ACS.

## Results

During the initial six-month pre-implementation study period, 7,335 adult patients had both troponin and CK-MB orders placed in the ED, compared with 736 in the post-implementation period (90% decrease). There was a 19.1% increase in troponin ordering (Table 1).

**Table 1:** Percentage of Patients with Negative Troponin and Positive CK-MB Discovered to Have ACS or Newly Diagnosed CAD ACS: acute coronary syndrome; CAD: coronary artery disease; CK-MB: creatine kinase-muscle/brain

Variable	N	%
Total patients	506	-
ACS diagnosed	3	0.60%
New CAD diagnosed	4	0.80%
Total ACS/new CAD diagnoses	7	1.40%

Of the 7,355 patients, 506 (6.9%) had a “negative” troponin at least three hours after symptom onset and a “positive” CK-MB result. As per our ED policy (based on ED literature [2,6,7], such patients were deemed not to have ACS (i.e., ACS was “ruled out.”). Of these 506 patients, 499 (98.6%) did not have a diagnosis of either ACS or new diagnosis of CAD during their ED and/or hospital stay; seven (1.4%) had either an ED or hospital discharge diagnosis of either ACS (3/7 patients [overall 0.6%]) or a new diagnosis of CAD (4/7 patients [overall 0.8%]) (Table 1). No patients had any significant dysrhythmic or hemodynamic event during their hospital stay; all were discharged alive (Table 2). Lastly, of the 265 positive cases with a positive CK-MB Index (+CK-MB-I), only two (0.8%) had a diagnosis of ACS or a new diagnosis of CAD (Table 3).

**Table 2:** % of Patients with Negative Troponin and Positive CK-MB Index Discovered to Have ACS or Newly-diagnosed CAD ACS: acute coronary syndrome; CAD: coronary artery disease; CK-MB: creatine kinase-muscle/brain

Variable	N	%
Total patients	265	-
ACS or new CAD diagnoses	2	0.8%

**Table 3:** # of Troponin and CK-MB Orders Pre- vs. Post-Implementation CK-MB: creatine kinase-muscle/brain; eGFR: estimated glomerular filtration rate

	Pre-implementation	Post-implementation	Change	% Change
Total combined CK-MB/troponin orders	7,335	736	-6,599	-90.0%
Troponin				
# of troponin orders	8,694	10,354	1,660	19.1%
Cost/test	$2.054	$2.054		0.0%
Total troponin cost	$17,857.48	$21,267.12	$3,409.64	19.1%
CK-MB				
# of CK-MB orders	7,421	831	-6,590	88.8%
# of “justified” CK-MB orders (eGFR <15 mL/min)	186	68	-118	-63.4%
# of “unjustified” CK-MB orders (eGFR ≥15 mL/min)	7,235	763	-6,472	-89.5%
Cost/test	$1.054	$1.054		0.0%
Total “unjustified” CK-MB cost	$7,625.69	$804.20	-$6,821.49	-89.5%

Owing to an increased number of troponin tests performed, the direct troponin processing expenses increased by $3,409.64. However, the reduction in CK and CK-MB order volume resulted in cost savings of $2,938.29 and $6,821.49, respectively, for a total savings of $9,759.78. Combining increased troponin expenses and decreased CK/CK-MB expenses yielded a net annual savings of $12,700 (Table 4). 

**Table 4:** Six-Month Cost Savings, Pre- vs. Post-Implementation CK: creatine kinase; CK-MB: creatine kinase-muscle/brain

Test	Cost Difference Pre- vs. Post-implementation
Troponin	$3,409.64
CK-MB	-$6,821.49
CK	-$2,938.29
Total	-$6,350.14

Confounding variables such as ED volume, volume of chief complaints for which cardiac biomarkers would likely be ordered (e.g., chest pain/shortness of breath), gender, and age were analyzed and not found to account for the decrease in CK/CK-MB order volume from 2017 to 2018 (Table 5).

**Table 5:** Confounder Analysis of Alternate Explanations for Decrease in CK/CK-MB Ordering CK: creatine kinase; CK-MB: creatine kinase-muscle/brain

	Pre-implementation	Post-implementation	% Change
Total # ED visits	49,633	50,564	2%
Total # ED visits w/ chief complaint of chest pain or shortness of breath	4,653	4,820	4%
# female patients	29,905	29,446	-2%
Mean age (years)	51.9	53.8	4%

## Discussion

Adoption of the new ED ACS evaluation guideline, which restricted CK-MB use in favor of a high-sensitivity troponin T strategy, was not associated with missing significant ACS events. During the six-month post-implementation period, only 6.9% of patients had a negative troponin with positive CK-MB. Of these, only 1.4% were diagnosed with either ACS or newly-diagnosed CAD; none died or had a significant complication while hospitalized. 

The new guidelines were also associated with a 90% reduction in the number of CK and CK-MB tests ordered. However, there was a 19.1% increase in troponins ordered, likely due to the introduction of high-sensitivity troponin two months after the CK-MB testing guidelines were implemented. Owing to this newer generation troponin T’s high sensitivity, up to one-third of samples result in intermediate/indeterminate values [4], in which a result is neither overtly “negative” (undetectable, or within the 99th percentile of the reference population) nor above the institution’s definition of an “elevated” result (in our case, >51 ng/L). At our institution, a value was considered intermediate/indeterminate if between 6-51 ng/L. To determine whether such a result may truly represent ischemia, our ED policy mandated a repeat troponin at least 1 hour after the initial troponin was drawn. Consequently, many patients who, prior to the adoption of high-sensitivity troponin, would have had only one (non-high-sensitivity) troponin sent, had repeat high-sensitivity troponin orders because their initial high-sensitivity troponin was in the intermediate/indeterminate range. Had our institution not transitioned to higher-cost high-sensitivity troponin ($2.054/unit) from standard troponin ($1.65/unit), and had the rate of troponin-ordering increased solely proportionate to the rate of ED visit increase (2% year-over-year) rather than increase 19.1% (likely due to the transition to high-sensitivity troponin), then the total six-month direct costs on troponin testing would have been $14,632 instead of $21,267.12, and annual direct cost savings would have been $18,945.80 instead of $12,700. 

This study is one of few that evaluated the safety of removing CK-MB and replacing it with troponin [10]. Other studies have shown CK/CK-MB does not add value to the determination of myocardial injury, and the correlation between CK-MB and the reduction of mortality is not statistically significant [11-13]. Eliminating CK/CK-MB from the diagnosis of ACS does not negatively impact the patient and is not associated with significant missed diagnoses, but is associated with significant savings. A study at John Hopkins Bayview Medical Center estimated a reduction of 50,000 tests and $1 million dollars when troponin was ordered alone, without CK/CK-MB, and no more than three times for the diagnosis of ACS [14]. CK/CK-MB orders add extraneous, unnecessary costs that can be avoided with the usage of the enhanced and superior high-sensitivity troponin T biomarker. Given its low specificity (40%) [15], routine CK-MB testing will yield many “false positive” results (elevations caused by non-cardiac causes). This may trigger significant downstream inconveniences, costs, and possible risks to patients, including hospitalization, specialty consultation, coronary calcium CT, echocardiogram, cardiac stress test, and coronary artery catheterization [16]. Cardiac catheterization has a complication rate of 1%, including vascular access hematoma, pseudoaneurysm, arteriovenous fistula, coronary artery dissection, and thrombosis and embolism [17]. A recent article of surveyed physicians found cascades of care after incidental findings to be common (90% of respondents), caused physical harm (15.6%), and financial burden (57.5%) and caused wasted time and effort (69.1%), frustration (52.5%), and anxiety (45.4%). One-third stated the test revealing their most recent incidental finding that triggered a cascade may not have been clinically appropriate [18].

This study has several limitations. First, the study was conducted at a single site and therefore might not be representative of the experiences of other institutions that have de-adopted routine CK-MB testing for ACS. Second, the study was done within a limited time frame; remote ACS events would not have been identified. Third, this study studied the behavior change of not ordering CK-MB for only a few months after introduction of the new guidelines; therefore, we do not know whether this behavior change will persist or whether providers will return to ordering CK-MB routinely. Finally, we did not determine indirect cost savings from elimination of CK-MB through the avoidance of “false positives” and subsequent downstream cascade of hospital admissions, diagnostic tests, and procedures.

## Conclusions

This study suggests that withdrawing routine-use CK/CK-MB and CK-MB Index in the evaluation of ACS in favor of an AHA/ACC-endorsed troponin-only strategy does not miss a significant number of ACS cases and is associated with direct cost savings. The cost savings we identified likely underestimate true cost savings from avoided downstream consequences of “false positive” CK-MB results (e.g., hospitalization, specialty consultation, coronary calcium CT, echocardiogram, cardiac stress test, and coronary artery catheterization).
